# Functional characterization and topological modularity of molecular interaction networks

**DOI:** 10.1186/1471-2105-11-S1-S35

**Published:** 2010-01-18

**Authors:** Jayesh Pandey, Mehmet Koyutürk, Ananth Grama

**Affiliations:** 1 Department of Computer Science, Purdue University, West Lafayette, IN, USA; 2 Department of Electrical Engineering & Computer Science, Case Western Reserve University, Cleveland, OH, USA; 3 Center for Proteomics & Bioinformatics, Case Western Reserve University, Cleveland, OH, USA

## Abstract

**Background:**

Analyzing interaction networks for functional characterization poses significant challenges arising from the noisy, incomplete, and generic nature of both the interaction data as well as functional annotation of molecules. Network-based methods focus on interacting molecules (pairs or sets) occurring in close proximity to infer functional associations.

**Results:**

In this paper we perform a formal comparative investigation of the relationship between functional coherence and topological proximity in networks. We investigate the problem of assessing the coherence of sets of biomolecules (or segments thereof) taking into account functional specificity as well as the distribution of functional attributes across entity groups. We also propose novel measures of topological proximity that are more robust to noisy and incomplete interaction data.

**Conclusion:**

We derive the following results in this paper: (i) there exists strong correlation between functional similarity and topological proximity in various network abstractions, with domain interaction networks (DDIs) demonstrating higher correlation than protein interaction networks (PPIs); (ii) measures that quantify coherence among entire sets of proteins are superior to aggregates of known pair-wise measures; and (iii) random-walk based measures of topological proximity are better suited to existing interaction data. We validate our methods on diverse data, including experimentally and computationally derived PPIs and DDIs, as well as on sets of known biologically related groups of molecules.

## Background

Analysis of interaction data generated from high throughput experiments takes a network-centric view of functions of biological systems and the role of the underlying components. Recent advances in this area have focused on the development of computational tools for network-based functional annotation [1], identification of functionally coherent modules [2], and relationship between network structure and function [3,4], among others. Network proximity and connectivity are also shown to be effective in identifying proteins that are implicated in similar phenotypes [5].

In this paper, we comprehensively investigate the relationship between topological and functional modularity in the context of two network abstractions - protein-protein interaction (PPI) and domain-domain interaction (DDI) networks. Key to understanding the relationship between network topology and functional modularity are: (i) suitable measures for assessing the *functional coherence *(or similarity) of a group of entities with respect to each other, and (ii) measures for quantifying the *topological proximity *in a network with potential missing interactions and noise. To assess functional coherence, canonical libraries of molecular function, such as Gene Ontology [6], are typically used [7]. Since annotations for different types of molecular entities (*e.g.*, proteins or domains) are derived in different ways [8], they have different implications with respect to their specificity and frequency distributions. Consequently, an important challenge in assessment of functional coherence is the development of measures that are robust to variations in distribution as well as missing data. In recent work, we have shown that information-theoretic measures that are specifically designed to address these challenges are effective in capturing the relationship between the functional coherence and network proximity of pairs of proteins [9]. In this paper, we build upon existing methods for quantifying functional coherence and topological proximity through the following key results:

• We propose novel measures for assessing the functional coherence of a group of molecules (in contrast to pairs of molecules).

• We propose the use of information flow based modeling of topological proximity and connectivity in a network of interactions (in contrast to traditionally used interaction counts or shortest paths).

We elaborate on these contributions below.

### Functional coherence of a group of molecules

Traditional measures of functional coherence, including our own prior results [9], have largely focused on pair-wise distance measures. Generalizing from pair-wise measures to coherence measures for sets of molecules adds significant complexity. For example, in testing the hypothesis that functional modularity is related to connectivity in PPI networks, it is common to investigate the functional purity of groups of proteins that induce dense subgraphs in the network [10]. While these enrichment-based methods have been widely used, they provide common overrepresented GO terms in a given set. They do not, however, provide a measure for the homogeneity of underlying modules (sets). We show that simple extensions of pair-wise measures to group measures by averaging, taking the min, max, or other such associative operations result in sub-optimal set-coherence measures. We propose novel measures of homogeneity of entire protein sets and demonstrate their superiority over generalized pair-wise measures on known groups of homogeneous complexes as compared to a control of randomly generated protein sets.

### Information flow based topological proximity

Topological information is used to identify functionally related proteins using shortest paths or density of direct interactions [1,5]. However, evidence suggests that multiple alternate paths between functionally associated proteins are often conserved through evolution, owing to their contribution to robustness against perturbations, as well as amplification of signals [11]. Consequently, consideration of multiple paths between molecules in a network of interactions is likely to be more effective in capturing the functional association between these molecules. Furthermore, consideration of alternate paths may account for missing data and noise in PPI networks [12]. There exist many methods for the assessment of network proximity based on the multiplicity of paths between nodes, including effective resistance [13], commute distance [14], and random walk proximity [15]. In this paper, we adapt an abstraction that models information flow in the cell using random walks with restarts [16].

## Methods

Several methods have been proposed for assessing functional similarity of biological entities (genes, proteins, domains) [17-19]. Since the functional categories in which these entities are categorized are themselves interrelated through a taxonomy (*e.g.*, Gene Ontology), measures for similarity must consider the underlying taxonomy while comparing molecules in terms of their functional annotation [20]. Various approaches take into account different factors, including taxonomic distance, specificity/generality (rank in hierarchy) of common ancestors, and associated number of molecules for the functional terms being compared (statistical significance or information content). Since most molecules are associated with multiple functional terms, assessment of functional similarity between two molecules poses the additional challenge of evaluating the similarity between two *sets *of terms, as opposed to a *pair *of terms. In [9], we developed an information theoretic measure for computing similarity of two sets of terms associated with a pair of molecules. We show that our measure is superior to other composite measures computed by applying associative operators (average, max, etc.) to pairwise term similarity measures.

In this paper, we generalize and extend our results to quantify the functional coherence (or similarity) of a *set *of biomolecules (as opposed to a pair). Since each molecule corresponds to a set of annotations, the problem is one of quantifying the coherence of a set of sets of terms. A straightforward approach to this would compute pairwise similarities of each pair of molecules in the set and to aggregate them using associative operators (min, max, average). Pairwise similarities (similarity of two sets of annotations) may themselves be computed using our information theoretic measure. An alternate approach to the problem, proposed in this paper, computes the coherence of the set of molecules without computing intermediate pairwise similarity scores. We show that the latter approach is strictly superior to the former in quantifying the coherence of a set of biomolecules. We validate this claim by applying our proposed measure, along with several other currently used measures to a test group of known functionally related proteins. We also apply the measures to randomly generated groups and identify measures that induce the greatest separation between the test and random groups.

Finally, in order to study the correlation between functional coherence and topological proximity in networks, we also need a measure for topological proximity. Traditional measures of topological proximity rely on the shortest path between two nodes. While this measure is more suited to well-curated and complete datasets, it is susceptible to missing interactions and noise. A single false positive or negative may lead to significant (erroneous) perturbation in shortest-path based measures. Measures based on random walks with restart [16], on the other hand, are more resilient to incomplete and noisy data. We consider both classes of measures of topological proximity, and evaluate their correlation with various functional similarity measures for both protein interaction (PPI) and domain interaction (DDI) networks. We show that a combination of random-walk based topological proximity and our similarity measure([9]) yield the strongest correlation between network proximity and functional coherence.

### Concepts and ontologies

Let *C *= {*c*_*i*_|1 ≤ *i *≤ *N *} be a finite partially ordered set of concepts. In terms of Gene Ontology (GO), these concepts represent the GO terms in the sub-ontologies (*i.e.*, molecular function, biological process, and cellular component). Without loss of generality, we refer to concepts as terms throughout this paper. Terms are related to each other through *is a *and *part of *relationships, such that *c*_*i *_→ *c*_*j *_denotes *c*_*i *_*is a/part of c*_*j*_. Note that, if *c*_*i *_→ *c*_*j*_, then the molecules associated with *c*_*i *_are also associated with *c*_*j*_, known as the *true path rule*. Based on these relationships, we define a binary relation over *C*, denoted by ≼. We say *c*_*j *_is an ancestor of *c*_*i*_, denoted by *c*_*i *_≼ *c*_*j *_if and only if either *c*_*i *_→ *c*_*j*_, or for some ℓ ≥ 1, there exist  for 1 ≤ ℓ ≤ 1 such that  for 1 ≤ ℓ < l, and  (*c*_*j *_is an ancestor of *c*_*i *_in GO hierarchy). Two terms *c*_*i*_, *c*_*j *_are comparable, denoted by *c*_*i *_~ *c*_*j*_, if either *c*_*j *_≼ *c*_*i *_or *c*_*i *_≼ *c*_*j*_. If *c*_*i *_and *c*_*j *_are comparable, then the shortest path between *c*_*i *_and *c*_*j *_is given by *L*(*c*_*i*_, *c*_*j*_) = *L*(*c*_*j*_, *c*_*i*_) = ℓ + 1 for minimum such ℓ.

We denote the set of ancestors of a term *c*_*i *_by *A*_*i *_= {*c*_*k *_∈ *C*|*c*_*i *_≼ *c*_*k*_}. Note that, not all ancestors of a term are comparable, since the GO hierarchy is a directed acyclic graph, as opposed to a tree. We represent the root term of GO with a terminal concept *r*, such that *c*_*i *_≼ *r *∀*c*_*i *_∈ *C*.

### Semantic similarity of terms

Semantic similarity measures quantify the similarity between two terms based on the underlying taxonomical relationships. The *information content *based measure of semantic similarity quantifies similarity between a pair of terms by taking into account the distribution of terms among molecules. Specifically, it rewards infrequent similar terms, over those that are frequent. Let *G*_*c *_be the set of molecules associated with term *c *in the available database, with *G*_*r *_being the set of all molecules. The information content of a term is defined as *I*(*c*) = - log2(|*G*_*c*_|/|*G*_*r*_|) [20]. Clearly, *I*(*r*) = 0, and as a consequence of the true path rule, *I*(*c*_*j*_) ≥ *I*(*c*_*i*_) for *c*_*j *_≼ *c*_*i*_. Then, the semantic similarity between two terms is defined as(1)

Here,  is said to be the *minimum common ancestor *of *c*_*i *_and *c*_*j*_.

Observe that this measure does not take into account the specificity of terms with identical common ancestors. This problem can be alleviated by normalizing the similarity between two terms by the self-similarities of the terms being compared, *e.g.*, by [21]. Note, this measure has a well defined maximum of 1 and offer bounded interpretation (ranging from 0 to 1) of Resnik's metric. We now generalize these term-similarity measures to set-similarity.

### Functional similarity of molecules

Biomolecules are generally associated with multiple molecular functions and often involved in multiple processes. Consequently annotations of molecules correspond to sets of terms, as opposed to individual terms. While assessing the similarity of sets of terms, we assume that the sets are non-redundant, *i.e.*, each set consists of terms that are not comparable. This can be easily enforced by ensuring that each branch in the hierarchy is represented by at most one term in each set. In GO, this involves considering only the *most specific annotations *associated with a gene, which provides a non-redundant representation of functional annotation. In this representation, the association between the gene and the ancestors of the most specific term is implied by the true path rule.

An important challenge in the assessment of the functional coherence of sets is that these sets are often incomplete (that is, for many molecules, some of their functions are unknown). Therefore, a reliable measure is one that rewards the abundance of similar terms in the terms, but does not penalize existence of unrelated terms in one of the sets, since the relation between these terms and the other set may be currently unknown. Simple associative measures that aggregate the similarity of pairs of terms in the two terms, such as average (*ρ*_*A*_) [17], maximum (*ρ*_*M*_) [22], or average of maximums (*ρ*_*H*_) [18] do not satisfy these properties [9].

Motivated by these considerations, in prior work, we extend the notion of minimum common ancestors to sets of terms, and generalize the concept of information content from a single term to a set of terms [9]. Let  be the minimum common ancestor set of term sets *S*_*i *_and *S*_*j*_, and ⊔ denote a generalized union operator that preserves non-redundancy by keeping the most specific terms. The similarity between two term sets is defined as the information content of the set of minimum common ancestors, *i.e.*,(2)

where  is the set of molecules that are associated with all terms in the set Λ(*S*_*i*_, *S*_*j*_). Note that *ρ*_*I *_also needs to be normalized with respect to self similarities, *i.e.*, *ρ*_*JC *_= 1/(*ρ*_*I*_(*S*_*i*_, *S*_*i*_) + *ρ*_*I*_(*S*_*j*_, *S*_*j*_) - 2*ρ*_*I*_(*S*_*i*_, *S*_*j*_) + 1)

### Functional coherence of modules

**Figure 1:**
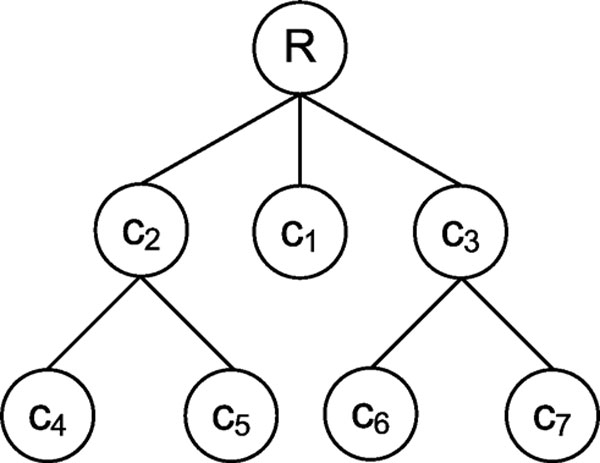
**Sample ontology**. *S*_1 _= {*c*_4_}, *S*_2 _= {*c*_4_}, *S*_3 _= {*c*_4_, *c*_6_}, *S*_4 _= {*c*_1_, *c*_6_}, *S*_5 _= {*c*_1_}, *S*_6 _= {*c*_6_}. Sample ontology and annotations. Each node of the hierarchy represents a term, each set represents a protein.

Let ℛ be a set of *n *molecular entities (genes, proteins, domains), with each entity being associated with a set of terms, *i.e.*, ℛ = {*S*_1_, *S*_2_, ..., *S*_*n*_}. We aim to develop a measure *σ*(ℛ) to assess the functional coherence of this set, such that a larger *σ *indicates more semantic similarity between the terms in sets *S*_1_, *S*_2_, ..., and *S*_*n*_. Without loss of generality, we call ℛ a module, since the objective here can also be considered as assessing the modularity of ℛ. We consider various measures to assess the functional coherence of a module, which are discussed below. In order to illustrate each measure, we use a running example based on the ontology shown in Figure 1. In the figure, let ℛ_1 _= {*S*_1_, *S*_2_, *S*_3_, *S*_4_} be a module that can be interpreted as a complex composed of two sub-complexes ℛ_2 _= {*S*_1_, *S*_2_, *S*_3_} (with the shared term *c*_4_) and ℛ_3 _= {*S*_3_, *S*_4_} (with the shared term *c*_6_), in which *S*_3 _"bridges" the two sub-complexes ℛ_2 _and ℛ_3_.

#### Average of pairwise information content

A straightforward way of computing set coherence is to compute the average of the pairwise *n*(*n *- 1)/2 set similarity scores [19,23]:(3)

In our running example, the average pairwise information content of the molecules in complex ℛ_1 _is given by *σ*_*A*_(ℛ_1_) = (*I*(*c*_4_) + *I*(*c*_4_)/2 + 0 + *I*(*c*_4_)/2 + 0 + *I*(*c*_6_)/4)/6 = 3/8, while that of sub-complex ℛ_2 _is given by *σ*_*A*_(ℛ_2_) = (*I*(*c*_4_) + *I*(*c*_4_)/2 + *I*(*c*_4_)/2)/3 = 2/3, given that *I*(*c*_4_) = *I*(*c*_6_) = -log_2 _(3/6) = 1. Bridged complexes get lower score than specialized complexes due to differences in sub-complex annotations.

#### Generalized information content

It is possible to extend the notion of the minimum common ancestor of pairs of terms to tuples of terms as . In the other words, the minimum common ancestor of a set of *n *terms is defined as the most specific among the terms that are common ancestors of all of *n *terms in the set. Then, for each n-tuple *c*_1 _∈ *S*_1_, *c*_2 _∈ *S*_2_, ..., and *c*_*n *_∈ *S*_*n*_, the functional coherence of these terms can be quantified as . Consequently, the minimum common ancestor set of *S*_1_, *S*_2_, ..., *S*_*n*_can be computed as

leading to a generalization of the information content based measure:(4)

In our running example, since *λ*(*c*_4_, *c*_1_) = *λ*(*c*_4_, *c*_6_) = *λ*(*c*_4_, *c*_1_, *c*_6_) = *r*, we have Λ(ℛ_1_) = {*r*}, thus the generalized information content of complex ℛ_1 _is *σ*_*I*_(ℛ_1_) = *I*(*r*) = 0. On the other hand, since Λ(ℛ_2_) = {*c*_4_}, we have *σ*_*I*_(ℛ_2_) = *I*(*c*_4_). As illustrated by this example, *σ*_*I *_is a rather conservative measure of functional coherence and it only rewards specialized modules in which all molecules share very similar functions.

#### Graph information content

We extend the graph information content measure proposed by Pesquita *et al. *[24]. The idea behind this approach is that, if a group of molecules are coherent, then the information content of the DAG induced by the intersection of ancestors is close to the information content of the DAG induced by the union of ancestors. In other words, defining  as the ancestor set *S*_*i*_, graph information content of set ℛ is defined as(5)

Observe that, if all molecules are annotated with the same set of terms, *σ*_*G*_(ℛ) would be equal to one, and zero if they have no common terms. Similar to *σ*_*I*_, a drawback of this measure is its sensitivity to outliers; that is, if a single molecule in the set is sufficiently functionally different it has a significant impact on the score. Indeed, in our running example, we have *σ*_*G*_(ℛ_1_) = *I*(*r*) = 0, while *σ*_*G*_(ℛ_2_) = (*I*(*c*_4_) + *I*(*c*_2_))/(*I*(*c*_4_) + *I*(*c*_2_) + *I*(*c*_6_) + *I*(*c*_3_)) = 1/2, given that *I*(*c*_2_) = *I*(*c*_3_) = -log_2 _(3/6) = 1.

#### Weighted information content

Complexes are functionally cohesive modules, but they are often composed of sub-complexes, each performing a specific part of the general function of the complex [25]. However, as illustrated by our running example, generalized information content (*σ*_*I*_) and graph information content (*σ*_*G*_) require all molecules to be functionally coherent with each other for the module to be considered coherent. In order to provide a more relaxed, and biologically motivated measure of functional coherence, we consider shared functionality between all combinations of molecules and weigh the information content of shared functionality by the number of molecules that contribute to the shared functionality.

Specifically, let  be the set of terms in the ancestor set of *S*_*i *_that are not shared with any other molecule in ℛ. Then, weighted information content of set ℛ is defined as the ratio of the information content of all terms that are shared in at least two molecules to the information content of all terms associated with at least one molecule in the set; that is:(6)

In other words, we consider all the partial DAGs () generated by each *S*_*i *_in ℛ. All the terms that are part of overlapping DAG correspond to shared information among those proteins. The numerator in the above equation corresponds to the information content of the overlapping DAG, while the denominator normalizes that score with total information of the combined DAG. In our running example, we have

*σ*_*W *_(ℛ_1_) = (3*I*(*c*_4_) + 3*I*(*c*_2_) + 2*I*(*c*_6_) + 2*I*(*c*_3_))/(3*I*(*c*_4_) + 3*I*(*c*_2_) + 2*I*(*c*_6_) + 2*I*(*c*_3_) + *I*(*c*_1_)) = 0.86 and *σ*_*W *_(ℛ_2_) = (3*I*(*c*_4_) + 3*I*(*c*_2_))/(3*I*(*c*_4_) + 3*I*(*c*_2_) + *I*(*c*_6_) + *I*(*c*_3_)) = 3/4, given that *I*(*c*_1_) = - log_2 _(2/6) ≈ 1.6 Since members of the module ℛ_1 _share all functions other than *c*_1_, this measure captures the coherence of the bridged module better than other methods. This method only penalizes for functions which are not shared by a member with rest of the module.

### Post-processing coherence scores

We now discuss how coherence scores are processed to make them comparable against each other for different module sizes and across various sub-ontologies.

#### Combination of sub-ontology scores

The scores discussed above can be based on any of the three sub-ontologies of GO. Since cellular component annotations are sparser than annotations of biological process and molecular function, we use the method proposed by Schlicker et al. [26]. For pairs of molecules, we combine the two coherence scores obtained from biological process and molecular function ontologies as:

where max *ρ*^(*BP*) ^and max *ρ*^(*MF*) ^are the maximum possible scores for biological process and molecular function, respectively. Module coherence scores (*σ*) are based only on biological process ontology.

#### Accounting for module size

In order to compare modules of different sizes, we normalize the functional coherence scores based on a background distribution that characterizes the coherence of modules of identical size. Specifically, for a given module ℛ, we generate a sufficiently large number of random modules of size |ℛ| and compute the functional coherence of each of these modules. Then, letting  denote the average functional coherence of these modules, we compute the size-adjusted coherence score of ℛ as .

#### Index of detectability

In order to compare various measures of functional coherence, we assemble a positive (test) group and a randomly selected (control) group of proteins. The positive set comprises of proteins that are known to be functionally related based on prior biological knowledge (*i.e.*, they are known to exist in complexes and perform related functions). Clearly, if we plot coherence values for samples from the test set and from the control set, we expect to see two distinct distributions - samples from the test group are expected to have higher coherence scores than those from the control group. The separation of the two distributions induced by each method indicates the fitness of the measure in quantifying coherence in sample sets, in terms of distinguishing coherent and arbitrary sets. This separation is quantified as:

which is proportional to the area under the binormal ROC curve [27]. Here, *T *and *C *denote the sets of test and control modules, respectively.

### Measure for topological proximity

The most commonly used measure of topological proximity is graph distance, where the distance between a pair of nodes in a connected graph is defined as the length of the shortest path between them. In the context of biological networks, there are several drawbacks to this measure. It is particularly susceptible to missing or incorrect data - *i.e.*, a single missing edge may reduce proximity significantly, alternately, a single false edge may increase proximity incorrectly [28]. Furthermore, this measure does not take into account the global structure and connectedness of the graph, with alternate paths between a pair of nodes.

Nodes connected to each other via disjoint paths are likely to be functionally closer than nodes that are connected via a single path. Indeed, evidence suggests that multiple alternate paths between functionally associated proteins are often conserved through evolution, owing to their contribution to robustness against perturbations, as well as amplification of signals [11].

To alleviate these drawbacks, we consider an alternate measure that captures the multi-faceted relationship between a pair of nodes [16]. This measure uses a random walk with periodic restarts to estimate the affinity between pairs of nodes. In this model, the random walk is initiated at node *i*, with neighbor transition probability proportional to edge weight, and at each step, the walk returns to source node *i *with probability *c*. The proximity of node *j *to node *i *is defined as the relative amount of time spent at node *j *by such an infinite random walk. It can be shown that the proximity of all nodes to node *j *can be computed iteratively as

Here, **W **is the stochastic matrix derived from the adjacency matrix of the network,  is the restart vector with  if *j *= *i *and 0 otherwise, and . Then, the proximity of node *j *to node *i *is given by . Repeating this procedure for all proteins, we obtain a matrix of network proximity scores for all pairs of proteins. Note, however, that this measure of proximity is not symmetric (proximity of *j *to *i *is not necessarily equal to the proximity of *i *to *j*). Therefore, we take the average of the two proximity values to compute the proximity between a pair of proteins. Using the proposed measures of functional coherence and the random-walk based measure for topological proximity, we quantify the relationship between topological proximity and functional coherence by computing the correlation of the resulting matrices.

### Materials

We obtain **protein interaction data **for *S. cerevisiae *and *S. pombe*, from the BioGRID database [29] version 2.0.51. We filter the dataset to obtain a set of physical interactions between proteins, *i.e.*, genetic interactions are removed based on experiment systems (*e.g.*, knockout experiments) mentioned on the BioGRID website. Integr8 [30] is used to map the proteins in the interaction dataset to their Uniprot names, keeping only those proteins that we can map to a Gene Ontology term using Integr8.

We obtain **domain interaction data **from the DOMINE database [31] version 1.1. This dataset is composed of known, as well as predicted domain interactions. Based on the source and quality of the data, we partition this dataset. **Struct **interactions are inferred from PDB entries of protein complexes and are collected from iPfam and 3did. **Comp-2 **interactions are predicted by at least two computational methods that infer domain interactions from protein interaction networks using techniques such as maximum likelihood estimation or from co-evolution of conserved sites in protein sequences. **HC+MC **interactions consists of high and medium confidence interactions (for details, please refer to [31]).

To test the **functional coherence of sets**, we obtain positive and random cases from GRIP [32]. GRIP generates positive cases from MIPS CYGD complex catalogue [33] by picking sets from known complexes. For wildtype cases, GRIP selects proteins at random. We generate a total of 16 datasets of which eight are made up of positive cases and eight are random. Each set consists 2000 sets of proteins (complexes), ranging from four to eleven proteins each.

**Gene Ontology Annotation **(GOA) [34] release 47.0 dated 2009/03/09 is used to obtain annotation information for Uniprot proteins. GOA combines manual and automated inferences of gene product annotations. The mapping of Pfam-A domains to their Gene Ontology functions is obtained from pfam2go http://www.geneontology.org/external2go/pfam2go released on 2009/03/04. We use only the Biological Process and Molecular Function sub-ontologies of Gene Ontology [6] version 1.550 for evaluation, since the coverage for the Cellular Component sub-ontology is relatively sparse.

## Results and discussion

We first compare the behavior of the molecular similarity metrics by examining their correlation with different topological proximity measures, and follow with a detailed look at their behavior on comprehensive PPI and DDI data. We then investigate the differences between PPI and DDI networks in terms of the relationship between network proximity and functional similarity using our generalized information content based metric. Finally we compare various measures for computing the functional coherence of sets. To evaluate similarity vis-a-vis proximity, we compute, for every pair of nodes in a network, the shortest distance between them, proximity for a given value of *c*, and various semantic similarity measures. Using these, we compute correlation of topological proximity metrics and functional similarity measures. As in [9] we normalize raw similarity scores to obtain a mean similarity score of zero and standard deviation of one. We create groups of pairs based on their topological proximity and compute average semantic similarity for each group.

### Topological proximity and semantic similarity measures

We first evaluate the proximity measure based on random walks. Since the parameter *c *can be varied to perturb the affinity between nodes, we first estimate an optimal value for *c*. We compute the proximity matrix for various values of *c*, ranging from 0.1 to 0.9, for the domain network *HC+MC*. We also compute the semantic similarity scores for different metrics - average of information content (IC) based term similarity (*ρ*_*A*_/*δ*_*I*_), average of self-normalized IC based term similarity (*ρ*_*A*_/*δ*_*JC*_), IC based molecule similarity (*ρ*_*I*_), and self-normalized IC based molecule similarity (*ρ*_*JC*_). We compute the correlation between these computed functional similarity scores and topological proximity. Semantic similarity is computed for the *biological process *(BP) and *molecular function *(MF) ontology separately, as well as by combining the two scores.

**Figure 2:**
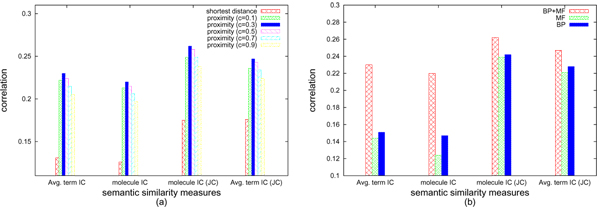
**Comparing topological metrics**. Comparison of different network distance measures in terms of their behavior with respect to semantic similarity metrics in **HC+MC **domain network, using the (a) for various values of *c *and shortest path (b) effect of ontologies.

As evident in Figure 2(a), for *c *= 0.3, we obtain the best correlation between the proximity matrix and any semantic similarity metric using combined BP and MF ontology. For further analyses, we use this value (*c *= 0.3) to compute the proximity matrix. In this network we also note that topological proximity (*c *= 0.3) has much better correlation with functional similarity than shortest path, for all similarity metrics. This validates our proposed use of random-walk based topological proximity measure. Indeed, this behavior follows our hypothesis that since proximity takes into account all paths from one node to another, two nodes connected in multiple ways are expected to be more functionally similar.

In Figure 2(b) we plot the correlation of topological proximity and semantic similarity measures using BP, MF and both ontologies. BP offers slightly better correlation than MF. In general, MF corresponds to a lower level property of a molecule directly related to its structure. BP is a higher level construct, related to the wider neighborhood in the network. Hence interacting molecules are more likely to belong to the same processes even if they have different functions. Finally the correlation obtained by combining the two ontologies is higher than taking them separately.

### Topological proximity and functional similarity in networks

**Figure 3:**
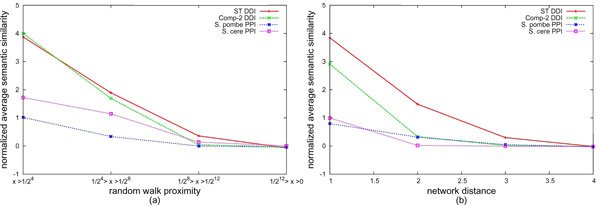
**Comparing PPIs and DDIs**. Comparison of various networks with respect to the relation between semantic similarity and (a) network proximity and (b) network distance.

Using the measure *ρ*_*JC *_by combining BP and MF ontology, we compare the relationship between functional similarity, random walk based network proximity (Figure 3a), and network distance (Figure 3b). We plot the normalized average semantic similarity, as in the previous case, for the PPI and DDI networks for various groupings of proximity values and shortest path distances. Each bin in Figure 3a is adjusted such that the number of pairs in each bin in Figure 3a is approximately equal to that in Figure 3b. As evident in the figure, the larger the proximity (between a pair of nodes) the (more) similar their functions. Conversely, lesser the distance between a pair of nodes, higher their similarity. Larger the slope between the groupings the better the measure performs (or dataset is) in grouping similar functioned molecules together. For both proximity measures, we find that DDIs have better functional similarity than PPIs, as also noted in [9]. Further, it is apparent that the relationship between functional similarity and topological proximity is stronger in computationally inferred DDI networks than that in PPI networks. Among the PPI networks, *S. cerevisiae*, which is the most completely annotated and studied, we observe stronger correlation between functional similarity and both proximity measures, compared to other PPIs.

Correlation of proximity measures and similarity in Figure 2a provides comparison of the curves in Figures 3a and 3b. Further comparison of Figures 3a and 3b indicates that the slopes of the curves are are generally higher for random walk based proximity, as compared to shortest path. Again, since proximity binds two nodes not just on topological distance but also on number of paths in between, only strongly correlated nodes have higher proximity values. These observations suggest that network proximity based on random walks is likely to be more relevant to, hence indicative of, functional coherence and modularity.

### Comparing measures for sets

We evaluate coherence measures for sets using index of detectability on sets with (known) functionally correlated protein and sets of randomly selected proteins. We compute functional coherence using the following measures: average of pairwise information content (using term *σ*_*A*_/*ρ*_*A*_/*δ*_*I *_and molecule *σ*_*A*_/*ρ*_*I *_based similarity), Generalized Information Content (*σ*_*I*_), Extended graph information content (*σ*_*G*_), and Weighted information content (*σ*_*W*_). As we observe in the previous section, since biological processes span wider neighborhoods, they are more likely to be shared in a module. For this reason, we compute the coherence score using only the biological process ontology. As the index of detectability is a measure of significance, we can plot a curve indicating the threshold corresponding to p-value < 0.05.

**Figure 4:**
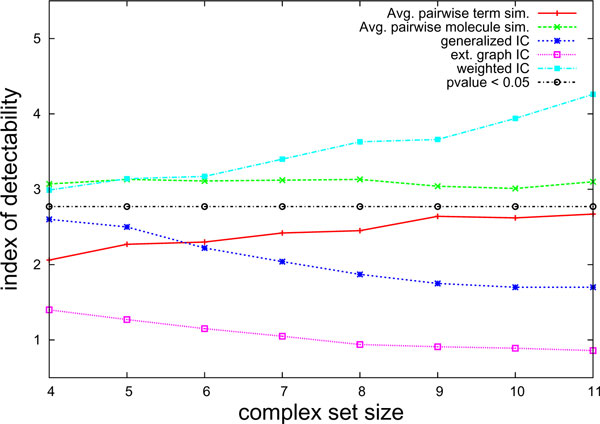
**Coherence in sets**. Comparison of detectability index for various coherence measures and complex sizes.

Figure 4 shows the index of detectability for various measures as the size of modules is varied from 4 to 11. We note that average of pairwise similarity based on molecule IC performs best from small modules, and that its performance remains steady as module size increases. Extended graph information content performs the worst and its performance decreases drastically as module size increases. As the module size increases, we expect the complex to be composed of sub-complexes with specific function, while the overall functionally shared among all molecules in this complex may be general. We see similar behavior in the generalized information content measure. The weighted information content based measure demonstrates improved performance as set size increases. This is because it can detect all shared functionality among sub-complexes that are parts of the entire complex, and have overlaps or bridges among them to carry out the biological tasks. Figure 4 also displays a curve indicating the threshold on index of detectability that corresponds to a statistical significance of *p *< 0.05 (according to normal distribution). This curve shows that only the weighted information content and pairwise molecule based similarity metric deliver significant performance in distinguishing known complexes and random sets of genes (*p *< 0.05), and the performance of the proposed measure increases with increasing complex size.

## Conclusion

We draw the following conclusions from our study: (i) our proposed measure of functional coherence of sets of entities (proteins, domains) is superior to other existing measures, (ii) we comprehensively study the relationship between functional coherence and topological proximity using suitable measures and derive formal conclusions for process- and function- based annotations, and (iii) we use our measures to study a range of PPIs and DDIs and establish the suitability of these abstractions to various kinds of analyses.

## Competing interests

The authors declare that they have no competing interests.

## Authors' contributions

JP developed the methods and performed all analyses. The three authors participated in the conception of the methods and the analysis. Together, the three authors wrote this manuscript.
